# Alpha and theta peak frequency track on- and off-thoughts

**DOI:** 10.1038/s42003-022-03146-w

**Published:** 2022-03-07

**Authors:** Jingyu Hua, Annemarie Wolff, Jianfeng Zhang, Lin Yao, Yufeng Zang, Jing Luo, Xianliang Ge, Chang Liu, Georg Northoff

**Affiliations:** 1 Center for Cognition and Brain Disorders, The Affiliated Hospital of Hangzhou Normal University, Hangzhou, Zhejiang China; 2 Institute of Psychological Sciences, Hangzhou Normal University, Hangzhou, Zhejiang, China; 3 Zhejiang Key Laboratory for Research in Assessment of Cognitive Impairments, Hangzhou Normal University, Hangzhou, China; 4 Department of Psychology, Faculty of Social Sciences, University of Ottawa, Ottawa, ON Canada; 5 Institute of Mental Health Research, University of Ottawa, Ottawa, ON Canada; 6 School of Psychology, Nanjing Normal University, Nanjing, Jiangsu China; 7 Center for Brain Disorder and Cognitive Science, Shenzhen University, Shenzhen, Guangdong China; 8 College of Biomedical Engineering and Instrument Science, Zhejiang University, Hangzhou, Zhejiang China; 9 Department of Neurobiology, NHC and CAMS Key Laboratory of Medical Neurobiology, School of Brain Science and Brain Medicine, and the MOE Frontier Science Center for Brain Research and Brain–Machine Integration, Zhejiang University School of Medicine, Hangzhou, Zhejiang China; 10 TMS center, Deqing Hospital of Hangzhou Normal university, Deqing 313200, China; 11 School of Psychology, Capital Normal University, Beijing, China; 12 Center for Psychological Sciences at Zhejiang University, Zhejiang University, Hangzhou, China; 13 Mental Health Centre, Zhejiang University School of Medicine, Hangzhou, Zhejiang China

**Keywords:** Cognitive neuroscience, Human behaviour

## Abstract

Our thoughts are highly dynamic in their contents. At some points, our thoughts are related to external stimuli or tasks focusing on single content (on-single thoughts), While in other moments, they are drifting away with multiple simultaneous items as contents (off-multiple thoughts). Can such thought dynamics be tracked by corresponding neurodynamics? To address this question, here we track thought dynamics during post-stimulus periods by electroencephalogram (EEG) neurodynamics of alpha and theta peak frequency which, as based on the phase angle, must be distinguished from non-phase-based alpha and theta power. We show how, on the psychological level, on-off thoughts are highly predictive of single-multiple thought contents, respectively. Using EEG, on-single and off-multiple thoughts are mediated by opposite changes in the time courses of alpha (high in on-single but low in off-multiple thoughts) and theta (low in on-single but high in off-multiple thoughts) peak frequencies. In contrast, they cannot be distinguished by frequency power. Overall, these findings provide insight into how alpha and theta peak frequency with their phase-related processes track on- and off-thoughts dynamically. In short, neurodynamics track thought dynamics.

## Abstract (web summary)

Hua et al. use EEG to track alpha and theta peak frequency alongside thought dynamics in healthy human participants. In doing so, they provide insight into how neurodynamics track thought dynamics.

## Introduction

Our thoughts show a high degree of dynamics in their often changing contents. These contents include single-thought contents like those related to a particular external task or stimulus, i.e., on-thoughts^1,2^. In contrast, if we do not focus on the task, our thoughts may wander around multiple more internal contents holding simultaneously (rather than sequentially) in our mind. These are off-thoughts and they reflect one type of mind-wandering^1,3–9^ which we here take as a ‘special case of spontaneous thought that tends to be more-deliberately constrained than dreaming’^6^. The difference between on- and off-thoughts in their dynamics, i.e., patterns of change, on the psychological level raises the following question: can the differential dynamics of on- and off thoughts on the psychological level be tracked by a corresponding dynamic on the neural level? Addressing this yet unresolved question is the goal of our investigation.

One influential hypothesis, the perceptual decoupling hypothesis^10,11^, assumes that off-thoughts are related to the decoupling of perception from external stimuli. The perceptual decoupling hypothesis concerns mainly the thought contents: if they are related to the presented stimulus or task, they are on-task and thus perceptually coupled^10,11^. If, in contrast, the thought content is not related to the stimulus or task, it is off-task and thus decoupled from the perception of the stimulus or task^10,11^. This leaves open whether, on the psychological level, on- and off-thought contents are not only associated with different contents, i.e., on- and off-task, but also with different numbers of contents, i.e., single or multiple with the latter holding simultaneously (rather than sequentially)^12^ in one’s mind. One would assume that on-thoughts are more likely related to single thought contents, i.e., one single item holding in one’s mind. While off-thoughts may be accompanied by multiple thought contents holding simultaneously, i.e., multiple items kept in mind at the same time. Hence, on- and off-thoughts may be associated with different numbers of thought contents indexing differential thought dynamics, i.e., patterns of change. Thus, characterizing the thought dynamics of on- and off-thoughts in terms of their number of associated thought contents, i.e., single vs multiple, was the first specific aim of our study.

Is the differential thought dynamic of on- and off-thoughts on the psychological level of on- and off-thoughts mediated by a corresponding dynamic on the neural level, that is, neurodynamic? Tracking thought dynamics in the brain requires high temporal precision in order to temporally relate the thought contents to the timing of the external stimulus. That can be achieved using EEG, which, unlike fMRI, provides high temporal resolution in the millisecond range. Various EEG studies reported different amplitudes in event-related potentials (ERP) like N100 and P300 (and other ERP’s) during on- and off-thoughts^2,13–18^. More dynamic oscillatory measures highlight the involvement of alpha^16,17,19–22^ and theta frequency bands^16,21,23–27^ during mind wandering. These findings raise the question whether dynamic changes in alpha and theta frequency power can track the thought dynamic of on- and off-thoughts with their potentially different number of thought contents, i.e., single vs multiple.

In addition to their power, neural activity in alpha and theta bands (and others) can be characterized by peak frequency sliding (FS) at specific points in millisecond time^28–31^. As shown in both computational modeling and human EEG, increases in alpha peak frequency (i.e., the alpha oscillation speeds up^30^), are directly related to increases in the input to the network and, on the more psychological level, to perception of specific contents^30^. This is consistent with findings showing that higher cognitive load during externally-oriented tasks lead to higher alpha peak frequency. Given that cognitive load is related to the number of items holding in one’s mind^32^, alpha peak frequency may be taken as an index of the number of thought contents, i.e., single vs multiple, holding simultaneously in one’s mind. We therefore hypothesize that alpha peak frequency, including its dynamic changes, i.e., sliding or change in peak frequency over time^30,31^, may provide a candidate measure to track the number of thought contents during especially on-thoughts as form of externally-oriented cognition.

Unlike the well-studied alpha peak frequency, the theta peak frequency has been less thoroughly investigated. Recent studies associate it with multiple more internally-oriented thought contents as in depressive rumination or working memory^31,33^. Moreover, theta peak frequency is supposed to stand in a specific relationship to alpha peak frequency with their harmonics facilitating processing of high cognitive loads with multiple thought contents^34^. Together, these findings let us hypothesize that the theta peak frequency and its dynamic changes, i.e., frequency sliding, may provide a suitable candidate measure to track the dynamics of specifically off-thoughts which, as form of internally-oriented cognition^35–37^, is presumably associated with multiple (rather than single) contents holding simultaneously in one’s mind.

**Fig. 1:**
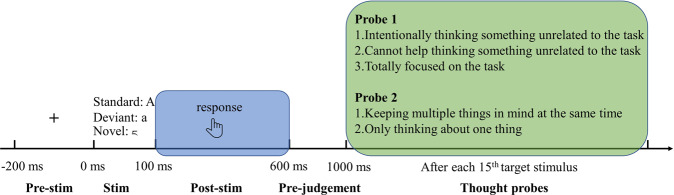
Experiment process and analysis schema. Experiment process: Each participant performed 1800 Sustained Attention to Response Task (SART) trials. In each trial, three types of stimuli were presented randomly for 100 ms after 200 ms fixation period: standard stimulus (upper case English letters), target stimulus (lower case English letters) and novel stimulus (letters from minority languages). Participants were requested to respond to the target stimuli by pressing the F key on the keyboard during the 900 ms blank window following stimulus (post-stimulus period). After each 15th target stimulus two thought probes were shown, participants were asked to answer the probe questions based on their types of thoughts (on- vs off-task and single vs multiple contents).

**Fig. 2:**
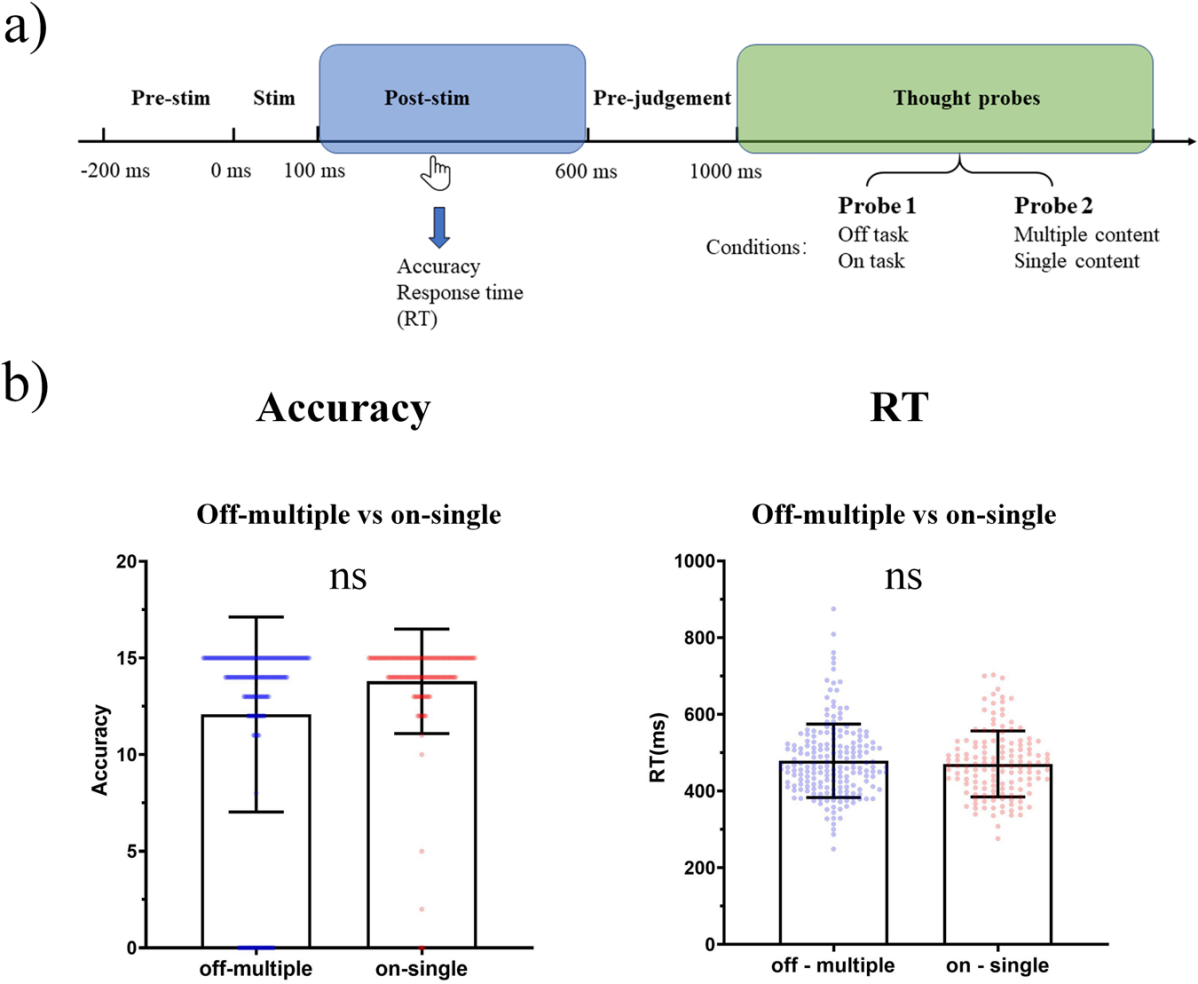
Behavioral analysis schema and results. **a** Analysis schema: Data analysis was trial-based across subjects. The accuracy, response time (RT), and probe answers were taken into behavioral analysis. All trials were divided into different conditions according to probe answers: off-thought vs on-thought based on the first probe, multiple-contents vs single-content based on the second probe, off-multiple vs on-single based on the combination of the first as well as second probe. GLMM and LMM were applied on accuracy and RT between conditions respectively. Chi square analysis was applied between two probes. **b** Results of GLMM and LMM on the accuracy and RT between off-multiple and on-single. There were no significant differences between off-multiple and on-single on accuracy or RT. The error bar means SD; ns: no significance.

**Fig. 3:**
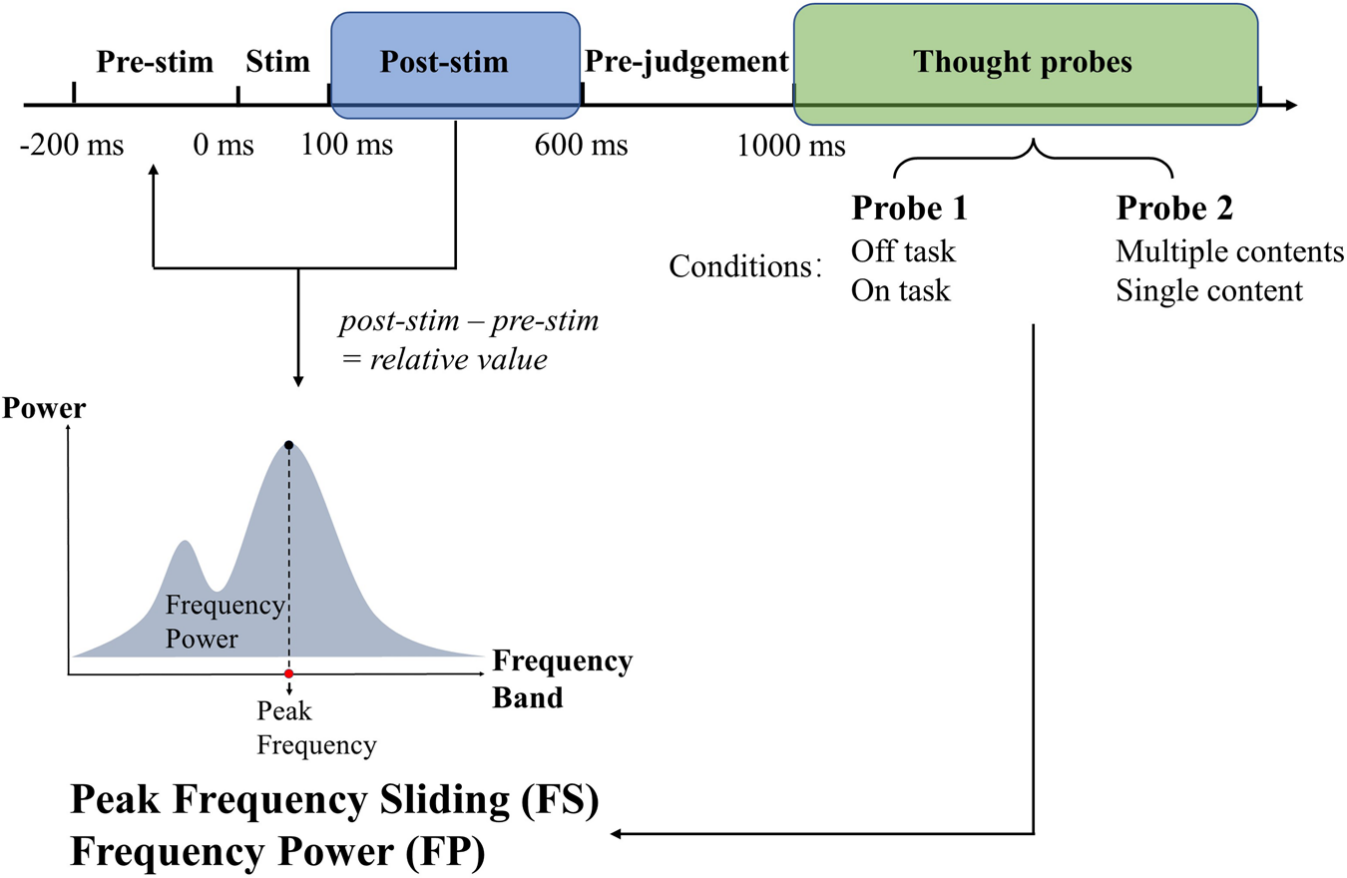
EEG analysis schema. EEG signals were first transferred to peak frequency sliding (FS) and frequency power (FP), then the average of pre-stimulus period (−200–100 ms) was subtracted from post-stimulus period (100–600 ms) to obtain the relative values. Alpha (8–13 Hz) and theta (5–8 Hz) post-stimulus relative values were compared between conditions, i.e., the different thought types, using LMM.

Using EEG, we apply a paradigm including neural, self-reported, and cognitive (accuracy) measures^9,38^ (see Fig. 1 for experimental overview), i.e., the Sustained Attention to Response Task (SART). We modified the standard SART in that we required participants to provide direct response to the target stimuli rather than, as in the standard version, holding their response. This allowed us to on-line explicitly specify the subjects’ number of thought contents as single (rather than multiple) as they only had to press the button when the target stimuli were presented (but no standard and novel tones; see methods for details). Unlike in standard SART, this allowed us to also use reaction time as a behavioral maker. Note that the response was only required to target stimuli but neither to neglect standard and novel stimuli. Such modified SART paradigm allowed us to measure the peak frequency sliding in the alpha and theta during the post-stimulus periods following the stimulus, i.e., post-stimulus interval where on- and off-thoughts occur non-confounded by and prior to the subjects’ judgment of the number of their thought contents (see Figs. 2a and 3).

Since our focus is on comparing different thought types in both their behavioral and neural correlates, we employed a way of analysing thought types, namely a trial-based analysis as distinguished from a subject-based analysis^39,40^. A trial-based analysis doesn’t average trials in each subject and does a comparison among conditions on the subject level, but takes all trials into account. For that purpose, we statistically calculated a linear mixed model as that allowed us to control for inter-subject variation^39,40^.

Our first major finding consists of showing that, psychologically, on-thoughts are associated with single thought contents whereas off-thoughts include multiple thought contents holding simultaneously in one’s mind. This suggests differential thought dynamics of on- and off-thoughts with respect to the number of their thoughts. The second major contribution is that, as postulated in our hypotheses, alpha and theta peak frequency track on-single and off-multiple thoughts in opposite ways: on-single thoughts show high alpha FS and low theta FS whereas off-multiple thoughts are tracked by the reverse pattern, i.e., low alpha FS and high theta FS.

Finally, it shall be noted that such distinction of the two types of thoughts could not be achieved when calculating alpha and theta power. Given that the only difference between alpha/theta peak frequency and power consists in the inclusion (peak frequency) and exclusion (power) of the phase angle^32^, we assume that phase-related processes take on a key role in tracking the differential thought dynamics of on-single and of-multiple thoughts. Mechanistically, that extends the current neuro-computational population-based model of input-peak frequency relationship^29,30^ to the neuro-cognitive level by relating neurodynamical changes in alpha and theta peak frequency to changes in thought dynamic, i.e., on-single and off-multiple thoughts. Broadly, neurodynamics track thought dynamics.

## Results

### Behavioral results—thought dynamics

Accuracy and response time (RT) are standard behavioral indexes of cognitive performance in mind-wandering like the SART paradigm^32,35^. First, we show no significant difference between off-multiple and on-single in both task-related accuracy and RT, as well as between off task and on task, multiple-contents and single-content (RT: off-multiple vs. on-single: *t* = −0.299; on task vs. off task: *t* = −0.353; multiple-contents vs. single-content: *t* = −0.454, CR: off-multiple vs. on-single: *z* = 0.376; on task vs. off task: *z* = 0.207; multiple-contents vs. single-content: *z* = 0.379, all *p* > 0.05, see Fig. 2b, Supplementary Fig. 1).

Secondly, according to previous studies, the state of ‘mind-wandering’ can be characterized by off-thoughts, i.e., ‘off task’ which was detected by probe 1 and ‘multiple content’, which was detected by probe 2^5,6^. We applied both independently and conducted fitting chi-square analysis to probe whether on- and off-thoughts can predict the occurrence of single and multiple thoughts. Our results show that the two probes are highly correlated (both *p* < 0.0001): when the first and second choices in probe 1 were chosen (off task), participants were much more likely to choose the first choice in probe 2 (multiple-contents).

### The neurodynamics of different thought states

We conducted a linear mixed model on event-related potential (ERP) components (N1 and P3) to probe whether these components are significantly different between the different kinds or types of thoughts (see “Methods” for details). The results show no significant differences between on-single and off-multiple thoughts in either N1 or P3 components (N1 on Fz: *t* = −1.744, *p* = 0.082; N1 on CPz: *t* = −0.930, *p* = 0.353; P3 on Fz: *t* = −1.539, *p* = 0.125; P3 on CPz: *t* = −0.561, *p* = 0.575; See Supplementary Fig. 4).

Next, we investigated peak frequency in a time-resolved way, i.e., frequency sliding (FS) in both alpha and theta bands which, following the protocol by Cohen^32^, is based on the phase angle (as calculated with Hilbert transform^32^). Additionally, we calculated the frequency power (FP) in both theta and alpha; the only difference of FP and FS is that the latter includes the phase angle, which is eliminated from the former by taking the modulus of the phase^32^. In short, peak frequency is phase-based, whereas frequency power is not.

**Fig. 4:**
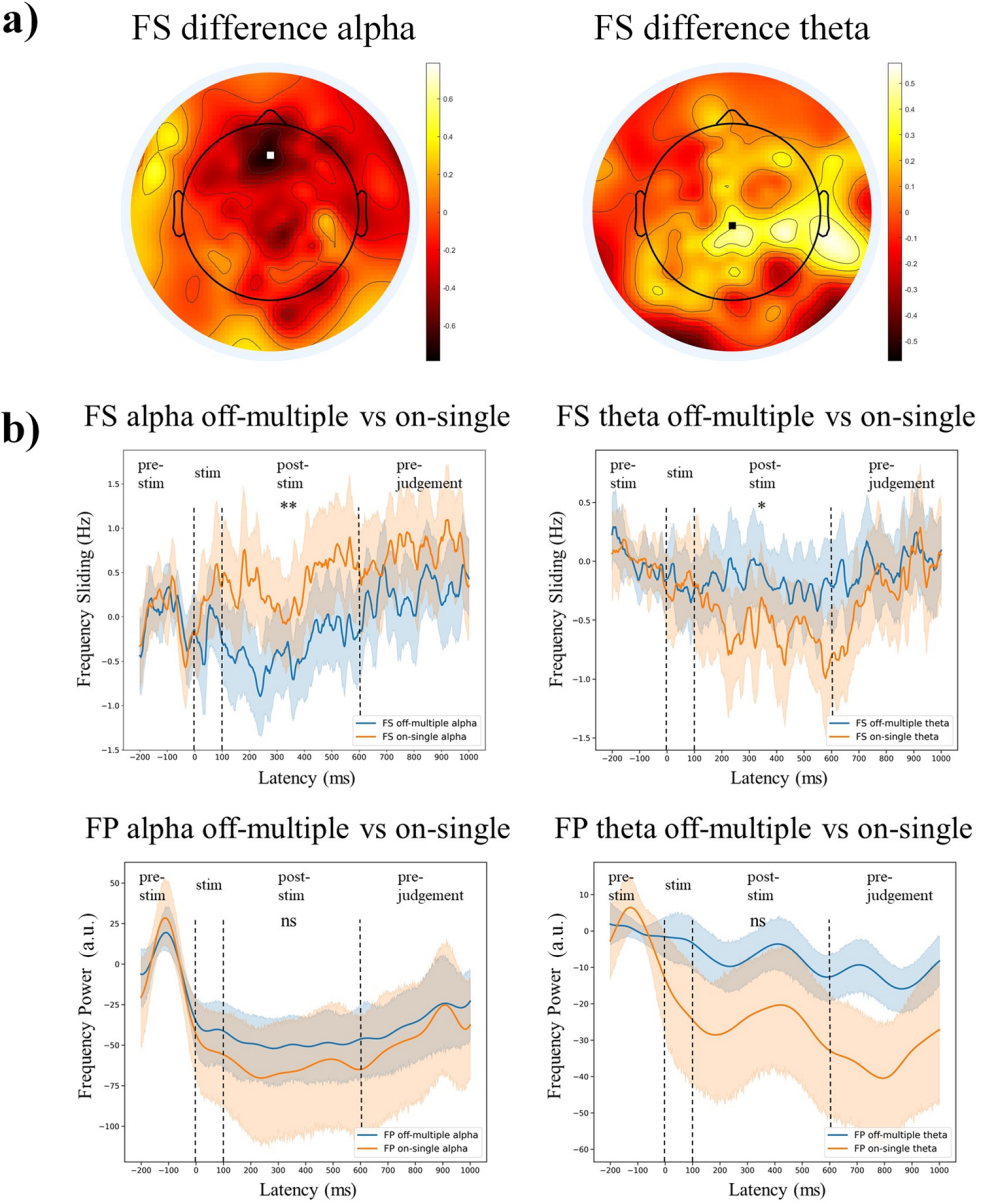
Frequency sliding (FS) and frequency power (FP) during different thought types. **a** The difference map of FS post-stimulus’ relative values. The difference map is calculated by subtracting on-single values from off-multiple values. According to visual inspection showing the strongest changes of FS, Fz was chosen for alpha FS and FP, CPz was chosen for theta FS and FP. **b** The time series of FS and FP relative values (subtracting the average of −200–0 ms from all time points) under each condition (off-multiple and on-single) with the results of LMM of post-stimulus relative values between off-multiple and on-single. Significant differences were found between off-multiple and on-single thoughts in both alpha and theta FS but not in FP. *p < 0.05; **p < 0.01; ns: no significance.

We first tested whether there were significant differences in peak frequency sliding (FS) and frequency power (FP) between the different thought conditions. In order to avoid carry-over effects of pre-stimulus values (−200–0 ms), we subtracted the FS and FP values in the task-free post-stimulus interval (where the spontaneous thoughts occur) from their respective values in the pre-stimulus period. According to the topographical differences between the FS of off-multiple and on-single thoughts, the electrode Fz was chosen for all subsequent analyses on alpha while CPz was chosen for all subsequent analyses on theta (Fig. 4a). The results show that FS in alpha band is significantly higher in on-task, single content and their combination (on-single thoughts) compared to off-task, multiple contents, and off-multiple thoughts. In contrast, FS in theta band showed the opposite results (off-multiple vs. on-single alpha: *t* = 2.964, *p* = 0.003; off task vs. on task alpha: *t* = 2.929, *p* = 0.004; multiple content vs. single content alpha: *t* = 2.720, *p* = 0.007; off-multiple vs. on-single theta: *t* = −2.077, *p* = 0.039; off task vs. on task theta: *t* = −1.891, *p* = 0.060; multiple content vs. single content theta: *t* = −2.049, *p* = 0.041; Fig. 4b; Supplementary Fig. 2). Unlike FS, FP did not yield such differentiation between the different thoughts. Only theta but not alpha frequency power showed significant difference between conditions (off-multiple vs. on-single alpha: *t* = −0.970, *p* = 0.333; off task v.s. on task alpha: *t* = −1.064, *p* = 0.288; multiple content vs. single content alpha: *t* = −0.443, *p* = 0.658; off-multiple vs. on-single theta: *t* = −1.870, *p* = 0.062; off task vs. on task theta: *t* = −1.520, *p* = 0.129; multiple content vs. single content theta: *t* = −1.644, *p* = 0.101; Fig. 4b, Supplementary Fig. 2).

In the next step, the correlations between FS (both alpha on Fz and theta on CPz) and the amplitudes in the ERPs (N1 and P3 on both Fz and CPz) were estimated. As shown in Table 1, no significance was obtained and all *R*^2^ values were rather low (<0.1, see Table 1).

**Table 1:** Results of the correlation between ERPs and FS.

		FS alpha (Fz)				FS theta (CPz)		
	Beta values	95%CI	*p* value	*R*^2^	Beta values	95%CI	*p* value	*R*^2^
N1 Fz	−0.049	−0.15 to 0.05	0.351	0.002	−0.042	−0.06 to 0.14	0.422	0.002
N1 CPz	−0.079	−0.18 to 0.02	0.127	0.006	0.037	−0.07 to 0.14	0.480	0.001
P3 Fz	−0.014	−0.12 to 0.09	0.782	<0.001	−0.027	−0.13 to 0.08	0.603	<0.001
P3 CPz	−0.003	−0.11 to 0.10	0.947	<0.001	−0.001	−0.10 to 0.10	0.990	<0.001

The correlations between FS (both alpha on Fz and theta on CPz) and the amplitudes in the ERPs (N1 and P3 on both Fz and CPz) were estimated. No statistical significance was found. Note. FS: frequency sliding; ERP: event-related potentials.

Following Rodriguez-Larios and Alaerts^34^, we analysed the degree of synchronization, i.e., harmony, of alpha and theta FS. The results show that on-single thoughts do not exhibit a significantly higher degree of alpha theta peak frequency harmonic than off-multiple thoughts (on Fz: *t* = 0.187, *p* = 0.852; on CPz: *t* = 1.028, *p* = 0.305; Supplementary Fig. 3).

Finally, to explore frequency ranges beyond theta and alpha, we also analysed the FS on delta (3 Hz–4 Hz) and gamma (30 Hz–40 Hz) on both Fz and CPz electrodes. The results show that neither delta nor gamma has significant difference between off-multiple and on single thoughts on both Fz and CPz electrodes (gamma Fz: *t* = 0.714, *p* = 0.476; gamma CPz: *t* = 0.913, *p* = 0.362; delta Fz: *t* = −1.060, *p* = 0.300; delta CPz: *t* = 0.407, *p* = 0.684; see Supplementary Fig. 6).

### The neurodynamic distances between different thought states

**Fig. 5:**
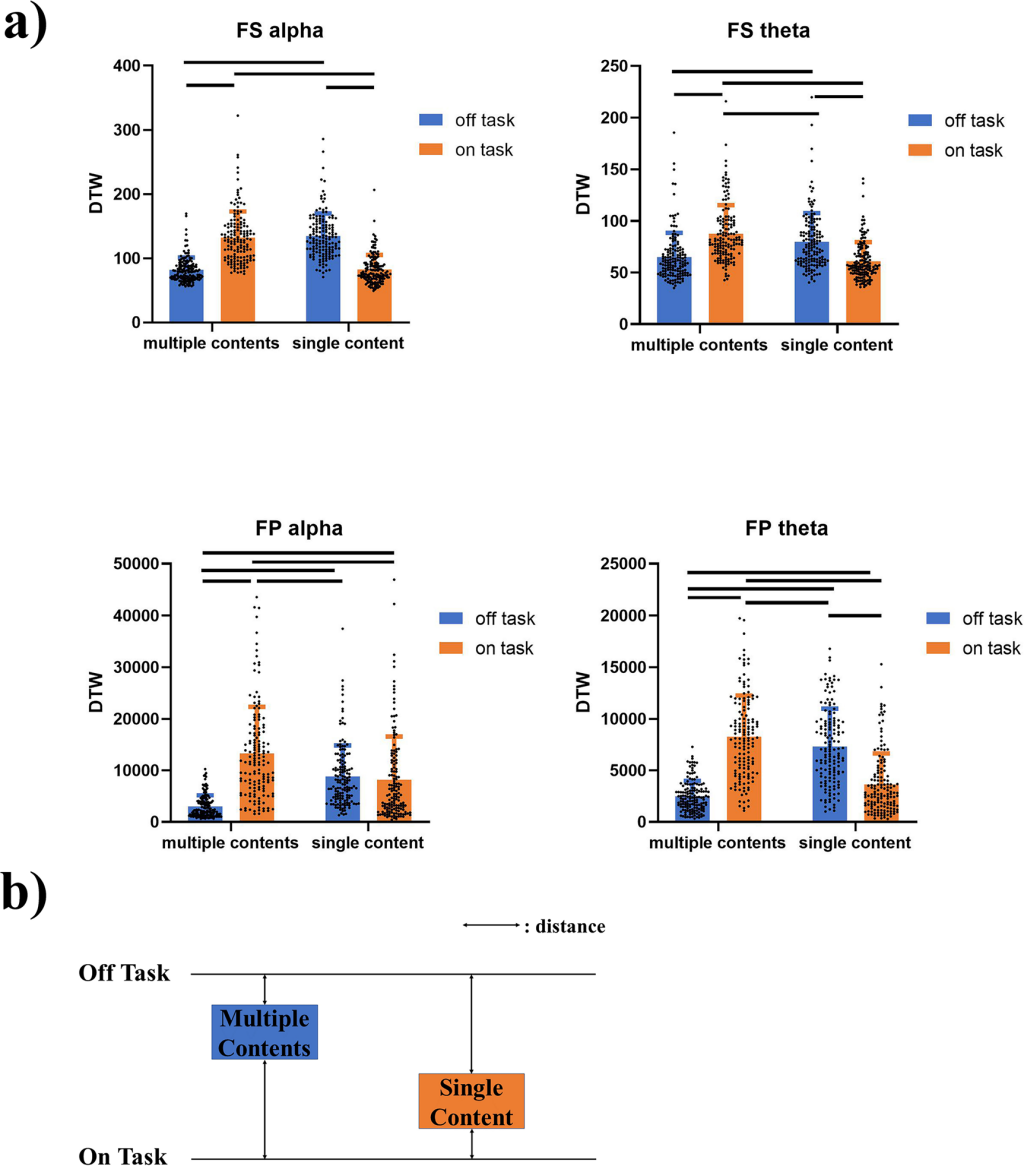
Results of FS and FP dynamic time warping (DTW). **a** The result of FS and FP DTW. The distances between off-task and multiple-contents, on-task and single-content are the smallest on both alpha and theta FS. The lines mean there’re significant differences between groups. **b** The schematic graph of the DTW result.

Following our behavioral data, off-thoughts are related to multiple contents while on-thoughts are rather associated with single content. In order to test their relationship on the neuronal level, we compared the alpha and theta FS timeseries’ associated with the different thoughts by utilizing dynamic time warping (DTW). The DTW is a measure for comparing the similarity between two time series. This allowed us to directly compare the temporal structure of two alpha/theta FS time series during on-thought FS with, for instance, the one during single content FS (and the same, analogously for all the comparisons among the four thought conditions). As is shown in Table 2, all interaction effects are significant (all *p* < 0.0001). The results show that in both alpha and theta peak frequency bands, the distances in the alpha/theta FS time series of on-thought vs single content are significantly lower than the ones of on-thought vs multiple contents and of off-thought vs single content (all *p* < 0.0001, Fig. 5a, Tables 2,  3). This result is the same for FP time series except FP alpha which does not show significant difference between off-single and on-single (all significant *p* < 0.0001; FP alpha off-single vs on-single: *p* = 0.8561 Fig. 5a, Tables 2,  3). The same holds for off-thought and multiple contents in both alpha and theta FS and FP. Hence, the DTW analysis supports the assumption of a close neurodynamic relationship of on-thoughts with single contents as well as of off-thoughts with multiple contents on the neuronal level (Fig. 5b).

**Table 2:** the ANOVA results of DTW.

	FS alpha		FS theta		FP alpha		FP theta	
Variance source	*F*	*p*	*F*	*p*	*F*	*p*	*F*	p
Probe 1	0.1247	0.7241	0.8343	0.3614	72.80	<0.0001	16.50	<0.0001
Probe 2	0.3687	0.5440	8.538	0.0036	0.47	0.4933	0.1499	0.6987
Probe 1 * probe 2	421	<0.0001	105.5	<0.0001	93.29	<0.0001	331.4	<0.0001

150 trials were randomly extracted and averaged across all trials at each timepoint to yield one alpha/theta FS (or FP) time series for each condition. Secondly, this process was repeated 150 times to get 150 DTW values for each pair of conditions (off-multiple, off-single, on-multiple, on-single). Then, two-way ANOVA was applied. The result show that all interaction effects are significant.

**Table 3:** Adjusted *p* values of multiple comparison on DTW.

	Off-multiple	Off-single	On-multiple	On-single
*FS alpha*				
Off-multiple		<0.0001	<0.0001	ns
Off-single			ns	<0.0001
On-multiple				<0.0001
On-single				
*FS theta*				
Off-multiple		<0.0001	<0.0001	ns
Off-single			0.0406	<0.0001
On-multiple				<0.0001
On-single				
*FP alpha*				
Off-multiple		<0.0001	<0.0001	<0.0001
Off-single			<0.0001	ns
On-multiple				<0.0001
On-single				
*FP theta*				
Off-multiple		<0.0001	<0.0001	0.0094
Off-single			0.0472	<0.0001
On-multiple				<0.0001
On-single				

After the two-way ANOVA, the Tukey’s multiple comparisons test was conducted upon DTW values.

### Different thought states show different degrees of uncertainty in their neurodynamics

**Fig. 6:**
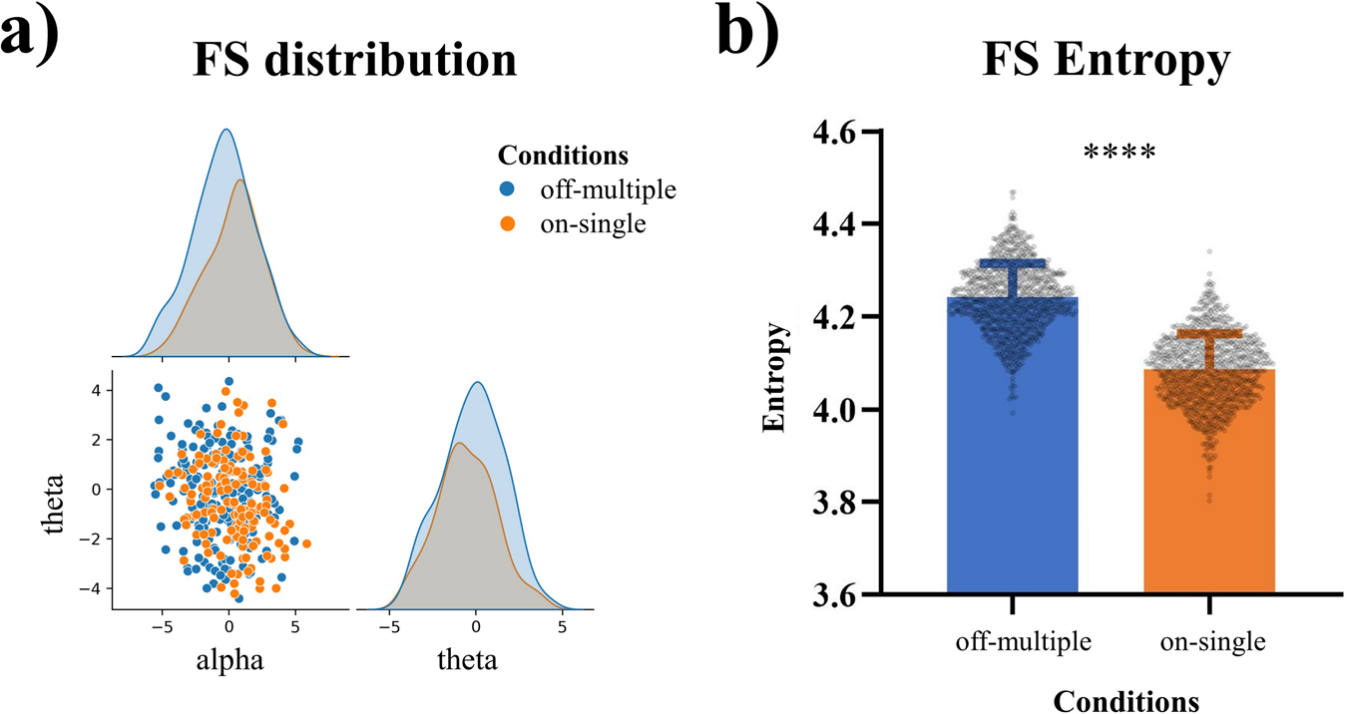
The joint distribution of FS alpha and theta and joint entropy (JE). **a** The joint distribution of alpha/theta FS (and FP). Off-multiple and on-single’s distribution could be discriminated in FS with overlaps. **b** The joint entropy (JE) results in FS. JE values for off-multiple thoughts are significantly higher than those for on-single thoughts. The error bar means SD. ****p < 0.0001.

In order to investigate the distribution of FS values in alpha and theta, joint entropy (JE), a measurement for describing the uncertainty of a set of variables, was applied to map their data distribution^36^. According to the joint distribution of alpha-theta FS, as measured by JE, off-multiple and on-single thoughts could be discriminated well by FS (*t* = 46.42, *p* < 0.0001). However, although the joint distribution of off-multiple and on-single thoughts is different from each other, the FS values of on-thoughts and single content are highly overlapped with the overall distribution or range of the ones for off-thoughts and multiple contents (Fig. 6). Moreover, we can see that the range (or variance) of the data distribution, i.e., JE of FS for off-multiple thoughts is significantly higher than the JE of on-single thoughts (Fig. 6).

Together, these results on joint entropy support again the good capacity of FS in distinguishing different thought types. Moreover, the results show that off-multiple thoughts exhibit a wider and more extensive data distribution, i.e., in FS than on-single thoughts. It shall be noted though that the data distribution of FS for on-single thought is included as more limited subspace with the more extended distribution of FC for off-multiple thought. That partial overlap of on-single FS values with the much larger range of off-multiple FS values suggest that the former may represent a subgroup of the latter.

## Discussion

The goal of our study was to investigate how the psychological dynamics of on- and off-thoughts with respect to the number of their contents (one vs many) is tracked by a corresponding neurodynamics of alpha and theta peak frequency. Our results revealed that, on the psychological level, on-thoughts are associated with single content, namely those related to the stimulus or task, while off-thoughts include multiple thought contents holding simultaneously in one’s mind. Neuronally, as measured in post-stimulus periods using EEG, the time course of on-single thoughts exhibited increasing alpha peak frequency and decreasing theta peak frequency (as measured by their frequency sliding) relative to pre-stimulus, while off-multiple thoughts showed the reversed pattern. Importantly, these thought-specific changes were only observed in the phase-angle based (Hilbert transform) alpha and theta peak frequency but not in their non-phase-based power (modulus); this suggests a key role of phase-related processes in tracking thought dynamic. Together, our study demonstrates that alpha and theta peak frequency including their phase-related processes are viable neurodynamic indexes to track the thought dynamics of on-single and off-multiple thoughts. As it will be outlined below, this extends and complements the current neuro-computational population level hypothesis of the proportional relationship of peak frequency to the degree of inputs^30,37^ to the neuro-cognitive level of thought dynamic.

On- and off-thoughts show a specific dynamic as they are related to different numbers of thought contents. We observe higher association of on-thoughts with single content while off-thoughts are characterized by multiple simultaneous contents during one and the same trial. That is further supported by our finding that, applying chi square analysis, on-thoughts can reliably predict single contents while, analogously, off-thoughts predict multiple contents. Finally, the cognitive relevance of the differential number of contents is supported by higher task-related accuracy during both on and single thoughts when compared to off and multiple thoughts.

Together, our findings suggest that on- and off-thoughts are not only distinguished in the relation of their contents to the respective external stimulus but also in their number of contents, i.e., single vs multiple. Especially, the occurrence of multiple contents holding simultaneously in off-thoughts suggests their close relationship to internally-oriented cognition: as they are not related to the external stimulus, they must be generated internally rather than externally. One may consequently hypothesize that multiple simultaneously occurring thought contents may reflect ongoing processes of different forms of internally-oriented cognition like self-relatedness^41,42^, mental time travel^43,44^, and emotional processes^41,45^. That, however, warrants future investigation. Accordingly, our observation of multiple thought contents holding during specifically in association with off-thoughts reveals a thought dynamic that may connect them closely to the various forms of internally-oriented cognition.

Can the thought dynamic of on-single and off-multiple thoughts be tracked by a corresponding dynamic on the neuronal level? We reveal that peak frequency of alpha and theta show different dynamic patterns during on-single and off-multiple thoughts. Specifically, relative to the stimulus/pre-stimulus period, the time course of alpha peak frequency (as measured by frequency sliding) show a post-stimulus increase during on-single thoughts. In contrast, alpha peak frequency decreased during the post-stimulus interval when subjects reported off-multiple thoughts in the subsequent judgment. Compared to alpha peak frequency, the theta peak frequency exhibited the reverse pattern with post-stimulus increase in off-multiple thoughts and decrease in on-single thoughts. Together, these findings strongly suggest direct relationship of neurodynamic changes of alpha and theta peak frequency to a specific pattern of thought dynamics.

The role of specifically peak frequency, i.e., alpha and theta for on- and off-thoughts is further supported by our findings in both ERP and power. Neither the N1 nor the P3, averaged at stimulus onset, could not distinguish on-single and off-multiple thoughts. Even more important, we also calculated the power in alpha and theta frequency by eliminating the phase-related information through applying the modulus of the phase (37). Replicating previous studies, we did observe power changes in the post-stimulus period in both alpha^16,17,19–21^ and theta frequency bands^16,21,23,26,27^. However, extending beyond the previous findings, we demonstrate that these power changes remain thought-unspecific, i.e., they were similar for on-single and off-multiple thoughts.

Given that the only difference between alpha/theta peak frequency and power consists in the inclusion (peak frequency) and exclusion (power) of the phase angle (37), we assume a special role of phase-related process in tracking the differential dynamics of on-single and off-multiple thoughts. Our findings thus support the assumption that the neurodynamics of specifically alpha and theta peak frequency (rather than their power) allows tracking the differential dynamics of on-single and off-multiple thoughts possibly through phase-related processes (as these were eliminated in the power).

What are the mechanisms by which peak frequency tracks on-single and off-multiple thoughts? Peak frequency is related to specific processes on both neuronal and cognitive levels. Neuronally, it is related to the neuronal input driving action potentials and population network activity: the more neuronal input, the higher population activity, and the higher the peak frequency^30^. At the same time, peak frequency in especially alpha is known to relate to single specific perceptual and cognitive contents: the higher the external perceptual or cognitive load, the higher alpha peak frequency^29,30,46^. Applied to our findings, this means that higher alpha peak frequency is most likely driven by increased neuronal input as related to the single external task-related contents of on-single thought.

How about off-multiple thoughts? Given that the off-thoughts are task-unrelated, i.e., off-task, the neuronal input must here be generated internally, that is, independent of the external input related to the stimulus theta^33,47,48^. That is indirectly supported by the fact that in off-multiple thoughts, the external input did not yield any increase in alpha peak frequency which, as our data in on-single thoughts suggest, are related to the neuronal input yielded by the external stimulus. Instead, we observed increase in theta peak frequency which, as we assume, may be related to internally-generated neuronal input during multiple-off thoughts. Being internally- rather than externally-generated, the neuronal input in off-multiple thoughts may thus be literally decoupled and independent of the external input just as hypothesized in the Perceptual decoupling hypothesis of mind-wandering^2,10,11,13,18^.

**Fig. 7:**
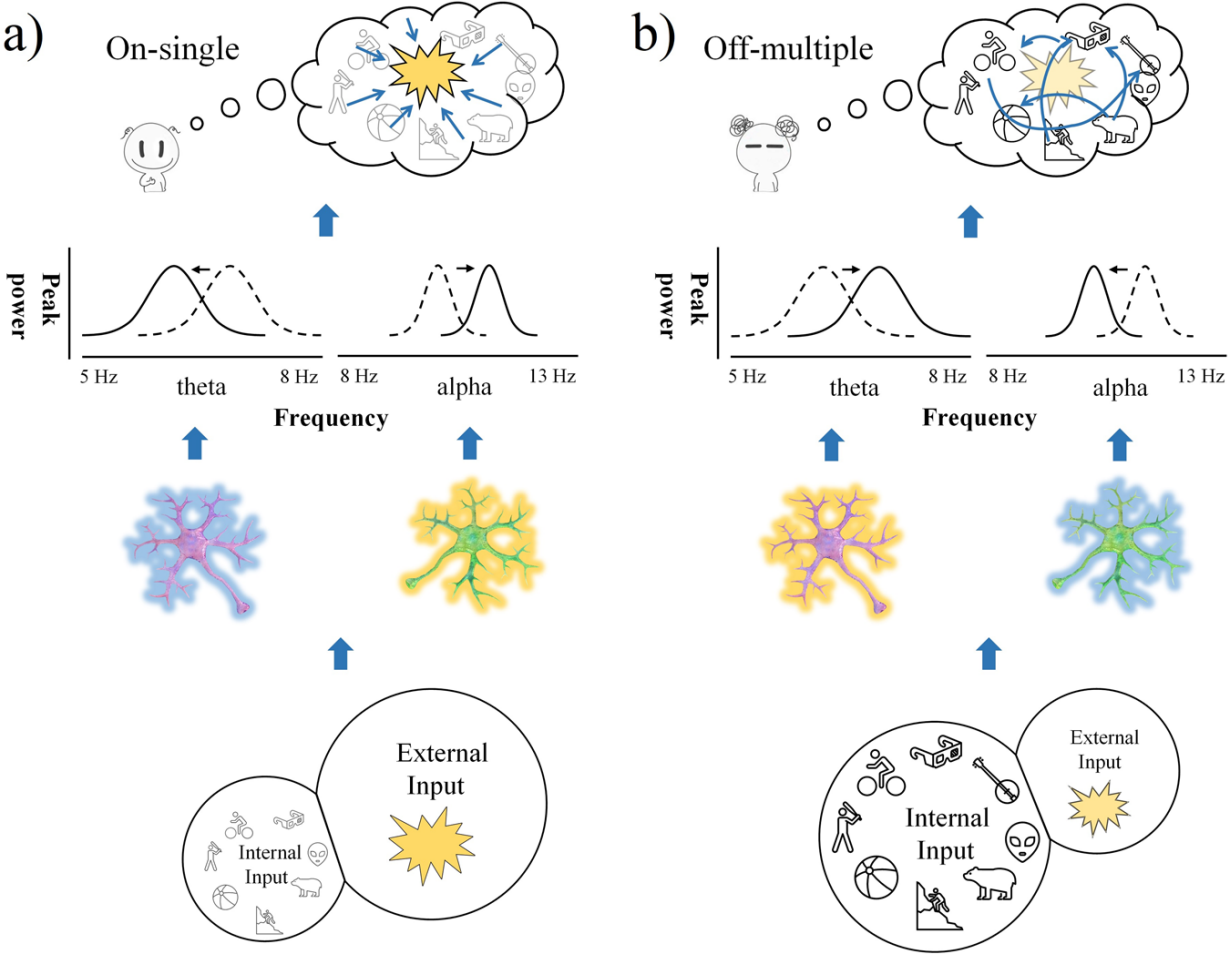
Schematic illustration of the proposed mechanisms mediating the relationship between neuronal input, alpha/theta frequency sliding and thought types. **a** - On-single thoughts. Here during external tasks, the single external input (yellow) dominates, activates external-neurons on the population level (lower level of graph), increases alpha peak frequency, decreases theta peak frequency (middle level), and leads to the cognition of on-single thoughts (upper level). **b** - Off-multiple thoughts. Here, multiple internal inputs dominate during external tasks (lower level) (blue), increase theta peak frequency, decrease alpha peak frequency (middle level), and are psychologically manifest as off-multiple thoughts (upper level).

Together, we assume that both on-single and off-multiple thoughts are mediated by neuronal inputs on the population level which, on the systemic level, is manifest in peak frequency (37). While the difference between the two thought types consists in distinct origins or sources of their respective neuronal inputs, i.e., external and internal^49^, which is mediated by peak frequency in distinct bands, i.e., alpha and theta^50,51^. (see Fig. 7).

Finally, our finding of thought-specific changes in alpha and peak frequency lends further support to and extends the different variations of process models of spontaneous thought proposed recently. These include (i) dynamic models which explains ‘mind-wandering’ in two dimensions of deliberate constraint and automatic constraint^6,52,53^, (ii) process model of spontaneous thought where ‘mind-wandering’ is represented by its off-task contents^54^, (iii) process-occurrence framework where task-related ‘mind-wandering’ is conceived as an assemble of different experiences and thoughts^1,4,55^, and (iv) the Spatio Temporal Theory of spontaneous Thought (STTT)^56^. Our results support and extend these models as we show that off-task thought is associated with multiple contents. Showing relationship between neurodynamics and thought dynamics, our findings support especially the STTT including its recent extension by the assumption of temporal dynamics as shared feature or “common currency” of neuronal and psychological levels of thoughts^39,57^.

### Methodological limitations

We applied a modified version of the SART that inserted a post-stimulus period with an immediate judgment on thought probes (rather than holding the judgment). This allowed us to directly test the neuronal changes in peak frequency prior to the single-trial judgment. While we tested for the relative changes in alpha and theta peak frequency during the post-stimulus interval compared to the stimulus/pre-stimulus periods, we can still not rule out carry-over effects from the latter to the former. These are rather unlikely, though, given that we observed changes in the neurodynamics of alpha and theta peak frequency in specifically the post-stimulus interval.

Moreover, due to the fact that the pre-stimulus period was rather short, we remain unable to investigate the impact of pre-stimulus changes on the post-stimulus interval. This may be necessary in the future though given recent studies that show how pre-stimulus changes modulate stimulus-related and post-stimulus activity^58,59^. This may be of strong interest as we assume that multiple contents may also be mediated during the pre-stimulus activity period.

Further more, as we had only 12 thought probes for each subject to avoid too much interruptions and only the trials prior to each thought probe were taken, only 12 trials for each subject came into the analysis. These 12 trials were further divided into different groups (i.e., thoughts) according to the thought probes answers just after them. Thus the number for each subject under each condition is low. Although the LMM and GLMM were conducted, the low trials number may still be a limitation of this paper. Experiments with more trials will be conducted in the future.

### Conclusion

We demonstrated that, on the psychological level, on- and off-thoughts are associated with different numbers of thought contents, i.e., single and multiple. Next, we showed how such thought dynamics are tracked by phase-based (Hilbert transform) alpha and theta peak frequencies rather than their non-phase-based (modulus) power. Measured by frequency sliding, alpha and theta peak frequency exhibited opposite temporal changes during on-single and off-multiple thoughts in our experiments.

Together, our results provide evidence that thought dynamics (the number of thoughts), during both internally- and externally-oriented cognition (off-multiple and on-single thoughts) is tracked by phase-related processes as measured with theta and alpha-peak frequency. Mechanistically, that extends the input-based population model of alpha- and theta peak frequency^32^ to the neuro-cognitive level by linking phase-related processes to thought dynamics. More generally, our findings suggest that temporal dynamics are realized in seemingly corresponding ways on both psychological and neural levels of thought, thus providing their hitherto missing link or “common currency”^39,57^. In short, we show how neurodynamics tracks thought dynamics.

## Methods

### Participants

Seventy right-handed adults participated in the study (32 female; age range = 18–29 years; mean age = 22.06 years, SD = 2.71 years). All had normal or corrected-to-normal vision and reported no neurological or psychiatric conditions that might affect performance. Of these, 9 were excluded as the data were not correctly recorded or got lost. Within these 61 subjects, 13 subjects whose 12 probe choices were all the same were excluded to ensure the validity of thought probes. After EEG preprocessing, 8 subjects were excluded for bad data quality (more than 50% epochs were excluded). Ultimately, 40 subjects’ data were entered into final analysis.

The methods were performed in accordance with relevant guidelines and regulations and approved by the research ethics committee of the Nanjing Normal University, School of Psychology, and the study was carried out with their permission. Verbal informed consent was obtained from each participant prior to study participation.

### Procedures

A three-stimulus Sustained Attention to Response Task (SART) was presented using E-Prime presentation software (version 2.10). We here used the standard SART for mind wandering^32,35,60^, which tests for subjects’ thought probes, combined with the presentation of different stimuli including standard, target (deviant), and novel (as in Oddball paradigms, see Stimuli Information in Supplementary Information for details). This paradigm served the purpose of explicitly specifying the subjects’ number of thought contents as single (rather than multiple) as they only had to press the button when the target stimuli were presented (but no standard and novel tones).

Subjects were instructed to press the ‘F’ on the keyboard in response to the target stimulus and ignore the other two types. All participants had learnt English for more than 10 years and were unfamiliar with the languages which were used for the novel stimuli.

The ratio of presentation of the three stimuli, standard: target (deviant): novel was 8: 1: 1. All three stimulus types were presented randomly. Participants first completed 50 trials without thought probes as practice. The testing phase then consisted of 6 blocks of 300 trials each. The stimulus was presented for 100 ms (stimulus period) after a fixation period of 200 ms (pre-stimulus period), followed by a blank window of 900 ms which, in the absence of judgment (see below), was labeled as the post-stimulus interval. The thought probes were presented at each 15th target stimulus, i.e., deviant stimulus; since the latter were presented randomly (relative to novel and standard), timing and occurrence of the thought probes could not be predicted by the subjects (reflecting pseudo-random distribution of the thought probes).

In the thought probes, participants had to choose the answers according to their thoughts following the task stimulus they had just seen previously. Two thought features were focused on in this study: the process (off-thought or on-thought) and the number of contents (multiple contents or single content). These two features were taken as the first and second probe respectively in fixed order. Moreover, according to the previous studies^6^, participants may deliberately move their attention off the task. This can be explained as a thought state where deliberate constraints predominate over automatic constraints. As this state is different from mind-wandering^6^, we explicitly asked subjects to decide between deliberate off-thought and automatic off-thought in the first probe. Taken together, the thought probes amounted to the following (see also Fig. 1a for the experimental design)

During the previous task, you were:

Probe 1:

1. Intentionally thinking something unrelated to the task.

2. Cannot help but think about something unrelated to the task.

3. Totally focused on the task.

Probe 2:

1. Keeping multiple things in mind at the same time

2. Only thinking about one thing

After the 3rd block an emotional induction video was played in order to induce either joy or neutral emotion. Videos come from the Chinese Emotional Visual Stimulus (CEVS) (neutral emotion induction: duration: 2 min 17 s, from the movie ‘Computer Repair’; joy induction: duration: 2 min 23 s, from the movie ‘a big potato’)^61^. Participants were asked to complete the Positive and Negative Affective Schedule (PANAS)^62^ before and after the emotional induction. However, as there was no significant difference of RT and accuracy between before and after emotional induction in all thought types by applying LMM and GLMM (all *p* > 0.05), emotion was not taken into consideration in this study. To increase the number of trials for both behavioral and neural analyses, we included all trials in all subsequent analyses.

Before the formal experiment, participants were firstly instructed into a training session which contained 40 standard stimuli, 5 target stimuli, 5 novel stimuli and 1 thought probes. Subjects were guided through the whole paradigm and thought probes, if they have any questions they could go through the training session again, and they could only go into the formal experiment if they have no further questions about the paradigm.

### Behavioral analysis

The analysis in this study was trial-based across subjects. The number of trials of conditions are shown in Table 4. The response time (RT) was the interval between stimulus onset and participants’ responses. Differences in RT between conditions, i.e., the four different thought types, were analysed using linear mixed model (LMM).

**Table 4:** The total trial numbers of each condition.

Probe 1	Probe 2	
	Multiple content	Single content
Off task	239	76
On task	7	158

The first and second choices in probe 1 were combined together as ‘off task’ while the third was taken as ‘on task’. The first and second choices in probe 2 were taken as ‘multiple contents’ and ‘single content’, respectively.

We also analysed the differences in accuracy of responses. As the accuracy is not a continuous variable, LMM is not appropriate. We first define accuracy as the number of correct responses between each of the two thought probes, and then calculated the generalized linear mixed model (GLMM) where Poisson distribution assumption was chosen.

It was mainly focused on thought states, i.e., off-task or on-task in this study in probe 1, so the first and second choices in probe 1 were combined together as ‘off task’. The probe 2 focused on the amount of thought content in participant’s mind. The contingency Table of the trial numbers of each condition were shown in Table 4, based on which chi square analysis was conducted to explore the relationship between probe 1 and probe 2.

### EEG recording and preprocessing

EEG data was collected from a 129-channel EEG system (HydroCel Geodesic Sensor Net, EGI System 300; Electrical Geodesic Inc., OR, USA) at a sampling rate of 1000 Hz and recorded with NetStation Software (Version 4.5.1, Electrical Geodesic Inc., OR, USA). Electrode Oz was used as the online reference. The impedance for all electrodes was kept below 50 kΩ while the data was recorded.

The data preprocessing was conducted using EEGLAB toolbox^63^ (http://sccn.ucsd.edu/eeglab/), which is a freely available, open source MATLAB-based package for EEG data analysis. The EEG signals were re-referenced off-line to the average of the left and right mastoids. The signals were band-pass zero-phase FIR filtered at 1–60 Hz with 46–54 Hz notch and were resampled (using antialiasing *resample* function) to 500 Hz. The data was then epoched from −200 ms to 1000 ms to the stimulus onset without baseline correction. The bad epochs during which the data quality is significantly worse than nearby epochs were rejected according to visual inspection. Participants with more than 50% of their epochs rejected were not included in subsequent analyses. All stationary artifacts, specifically eye movements (blinks and saccades), were reduced using Independent Component Analysis (ICA) and Principle Component Analysis (PCA) by the EEGlab toolbox.

### Statistical analysis

All analyses were trial-based across subjects. The total trial numbers of each condition, i.e., thought probes, are shown in Table 4. The trials with target stimuli which are just prior to thought probes were taken into analyses. After epoch-rejections, we have 286 trails for off-task, 156 trials for on-task, 227 for multiple-contents, 215 for single-content, 221 trials for off-multiple, 150 trials for on-single went into analysis. To ensure probe answers truly reflected participants’ thoughts, only the data from those who didn’t choose the same choices in subsequent probes across all trials were included in all other analyses. *T*-tests and ANOVAs were done in SPSS (version 21). The joint entropy analysis was done in python by using entropy_estimators toolboxe (version 0.0.1). As pointed out in the introduction, our primary focus was on comparing thought types irrespective of inter-subject differences. We, therefore, employed linear mixed model for statistical analyses as that allows to control for inter-subject differences^39,40^. The differences between conditions for both behavioral and EEG analysis were analysed by Linear mixed model (LMM) or generalized linear mixed model (GLMM, only for the accuracy analysis). All the LMM analyses treat random intercepts for participants as random effects. Both LMM and GLMM were done in R by using lmerTest package.

#### Definition of accuracy and response time (RT)

The probe questions were presented after each 15th target stimulus, thus between each two probes there were 15 responses. The accuracy was defined as the number of correct responses within these 15 responses. In behavioral analysis, the accuracy for each thought type was calculated by the answers to the thought probes following these 15 responses.

RT was defined as the time period between stimulus onset and participants’ response to target stimuli.

The differences of accuracy between conditions were analysed by the GLMM for which the Poisson distribution assumption was chosen. The differences of RT were analysed by the LMM. In both GLMM and LMM the conditions (off-thought vs on-thought, multiple content vs single content, off-multiple vs on-single) were taken as fixed effects and the random intercepts for participants were modeled as the random effect. The relationship between probes were analysed by Chi square analysis.

#### Event-related potential (ERP)

Two ERP components were identified, which were N1 and P3. The mean amplitudes of these components were taken within the following windows: N1 (0–200 ms), P3 (200–500 ms). The mean amplitudes were then compared between off-multiple and on-single on both electrodes Fz and CPz, respectively by applying the LMM. The conditions (off-multiple vs on-single) were taken as the fixed effect and the random intercepts for participants were taken as the random effect.

#### Peak frequency sliding (FS) and frequency power (FP)

Following our hypotheses (see above), we focused on alpha (8–13 Hz) and theta (5–8 Hz) frequency bands, measuring both their peak frequency change with frequency sliding (FS) and their power change (FP)^30,33,34,37^. According to the difference map of FS^64^, the Fz electrode was chosen for FS and FP in alpha and CPz was chosen for theta (Fig. 4a).

All preprocessed EEG data were first epoched to 1500 time points (−1000 ms–2000 ms) to avoid the edge effect. The FS and FP were calculated according to the method of MX Cohen^30^. The preprocessed data were first FIR bandpass filtered with 15% transition zone added to each edge of the filter range, then a Hilbert transform was done after which the phase angle timeseries was extracted. The FS is the first derivative of the phase angle timeseries and a median filter was applied in order to reduce the non-physiological noise^30^. For the FP, the analysis was the same as for the FS with only one difference: the modulus of the Hilbert transform was extracted rather than the phase angle timeseries. After FS and FP calculation, the average of pre-stimulus (−200 ms–0 ms) were subtracted from all time points (−200 ms–1000 ms) to get the values relative to the pre-stimulus period. The period from 600 ms to 1000 ms were taken as pre-judgement and excluded from analysis as in both off-multiple as well as on-single conditions more than 90% responses were made before 600 ms. Then the post-stimulus relative values (100 ms–600 ms) were extracted for alpha/theta FS and FP. Then the differences of FS and FP between the different thought conditions were analysed by applying the LMM as the analysis was trial-based. In the LMM modeling, the conditions (off-thought vs on thought, multiple content vs single content, off-multiple vs on-single) were taken as the fixed effect and the random intercepts for participants were taken as the random effect.

#### Dynamic time warping (DTW)

On the psychological level we tested whether on- and off-thoughts predict single and multiple thoughts. In order to probe the analogous prediction on the neural level, we used dynamic time warping (DTW). This was done to compare the time course of alpha/theta FS between the different thought types, i.e., on-single and multiple-off (and all other possible constellations).

DTW is a tool to compare different time series in terms of their mathematical distances such as Euclidean distance^65^. While DTW has previously been applied to EEG signals^65^, we here, for the first time to our knowledge, use DTW to compare the data from different frequency bands (alpha and theta) in the time domain, i.e., alpha/theta FS and FP. In this study, the time series being measured is −200 ms–1000 ms alpha/theta FS values. To compare the time course mathematical distances of alpha/theta FS (and FP) among conditions, firstly, 150 trials were randomly extracted and averaged across all trials at each timepoint to yield one alpha/theta FS (or FP) time series for each condition. This provided the basis for applying DTW. Secondly, this process was repeated 150 times to get 150 DTW values for each pair of conditions (off-multiple, off-single, on-multiple, on-single). Then, the two-way ANOVA was applied, after which Tukey’s multiple comparisons test were conducted (Fig. 5a, Table 3).

#### The determination of alpha-theta peak frequency’s ‘harmonic locking’

Different frequency bands usually represent different cognitive functions^66^. The synchronization between distinct rhythms is a core mechanism to integrate neural systems at different spatiotemporal scales^67,68^. A recent study demonstrated that a 2:1 harmonic relationship between alpha and theta peak frequency is related to their higher synchronization and more efficient cognitive performance in participants^34^. To do this analysis, the proportion of time-points in which the alpha–theta peak ratio equaled 2.0 (henceforward termed “harmonic locking”) was determined for each electrode, trial, and condition^34^. We then compared the proportions between off-multiple and on-single thoughts on electrodes Fz and CPz. We chose these electrodes to analyze the alpha and theta FS, respectively.

#### Joint entropy

Joint entropy is a measurement from information theory, it is a measure of the uncertainty of a set of variables (in our case 2 variables as alpha and theta peak frequency)^36^. The function of joint entropy is:$\,H\left(X,Y\right)=-\mathop{\sum}\nolimits_{x}\mathop{\sum}\nolimits_{y}P\left(x,\,y\right){{{\log }}}_{2}[P(x,y)]$, where *x* and *y* are particular values of *X* and *Y*, and *P*(*x*, *y*), is the joint probability of specific degrees in their values occurring together. Thus, joint entropy can be taken as a description of a joint probability distribution: the larger the entropy, the larger the uncertainty and lower the probability of specific values a particular variable can take on over multiple trials.

In this study, the joint entropy was used to describe the uncertainty of the changes in alpha/theta FS (and FP) during the post-stimulus interval (relative to the pre-stimulus period). The average of the post-stimulus period alpha/theta FS (and FP) relative changes in each trial were calculated for all trials, and then averaged across trials. The joint entropy was calculated on the joint distribution of alpha and theta values. Firstly, 150 trials were randomly chosen from each condition (off-multiple, on-single) by bootstrapping to calculate one entropy value for all these 150 trials. This process was repeated 1000 times. This allowed us to compare the joint entropy values between conditions (i.e., the different thought types) using independent *T*-tests (Fig. 6c). The joint entropy was computed by the entropy_estimators toolkit (version 0.0.1) available in python, which provides a tool to calculate the joint entropy of continuous multi-variables from the determinant of the multivariate normal distribution. Finally, to compare the differences between conditions for alpha/theta FS (and FP), the differences (calculated by the subtraction between the entropies of chosen conditions) were normalized to z-scores. The independent *T*-tests were then applied.

#### EEG analysis—statistical analyses

In the EEG analysis, All the ERP, FS, and FP analyses on alpha were done on the electrode Fz while for theta we took CPz according to the FS differences in the topographic maps (the difference between FS of off-multiple and FS of on-single, see Fig. 4a). For the ERP’s, the LMM were applied on two components comparing off-multiple and on-single: N1 (0–200 ms) and P3 (200–500 ms). The amplitude of these components was taken as the mean of each time periods. For FS and FP, the LMM was applied on the average of post-stimulus values relative to the pre-stimulus interval (100 ms–600 ms) between conditions (e.g., off-thought vs on-thought, Fig. 3). After that, the correlation was applied for probing the relationship of ERP components and FS. To do this, the FS and ERP values were first standardized by z-score within each subject, then the linear correlation was applied for all the trials. Furthermore, the peak frequency sliding (FS) and frequency power (FP) absolute values from −200 ms to 1000 ms were taken as a time series and the dynamic time warping (DTW) was applied to get the distance between conditions (Fig. 5). Independent *T*-test was also applied on FS entropy values to explore the difference of distributions between off-multiple and on-single. (Fig. 6). See the Supplementary Information for details of the analysis.

### Reporting summary

Further information on research design is available in the Nature Research Reporting Summary linked to this article.

## Supplementary information

Supplementary Information

Description of Additional Supplementary Files

Supplementary Data 1

Supplementary Data 2

Supplementary Data 3

Supplementary Data 4

Supplementary Data 5

Supplementary Data 6

Supplementary Data 7

Supplementary Data 8

Supplementary Data 9

Reporting summary

## Data Availability

The datasets generated during and/or analysed during the current study are available from the first and corresponding authors on reasonable request. Source data underlying Figs. 2 and 4–6, and Supplementary Figs. 1–5 can be found in Supplementary Data 1–9, respectively.

## Code availability

The scripts generated and used in the current study are available from the first or corresponding authors on reasonable request.
