# Supplementary material for: Alpha and theta peak frequency track on- and off-thoughts
Source: Commun Biol. 2022 Mar 7;5:209. doi: 10.1038/s42003-022-03146-w (PMC8901672; doi:10.1038/s42003-022-03146-w)
Supplement: Supplementary file 1 — Supplementary Information [file 42003_2022_3146_MOESM1_ESM.docx]

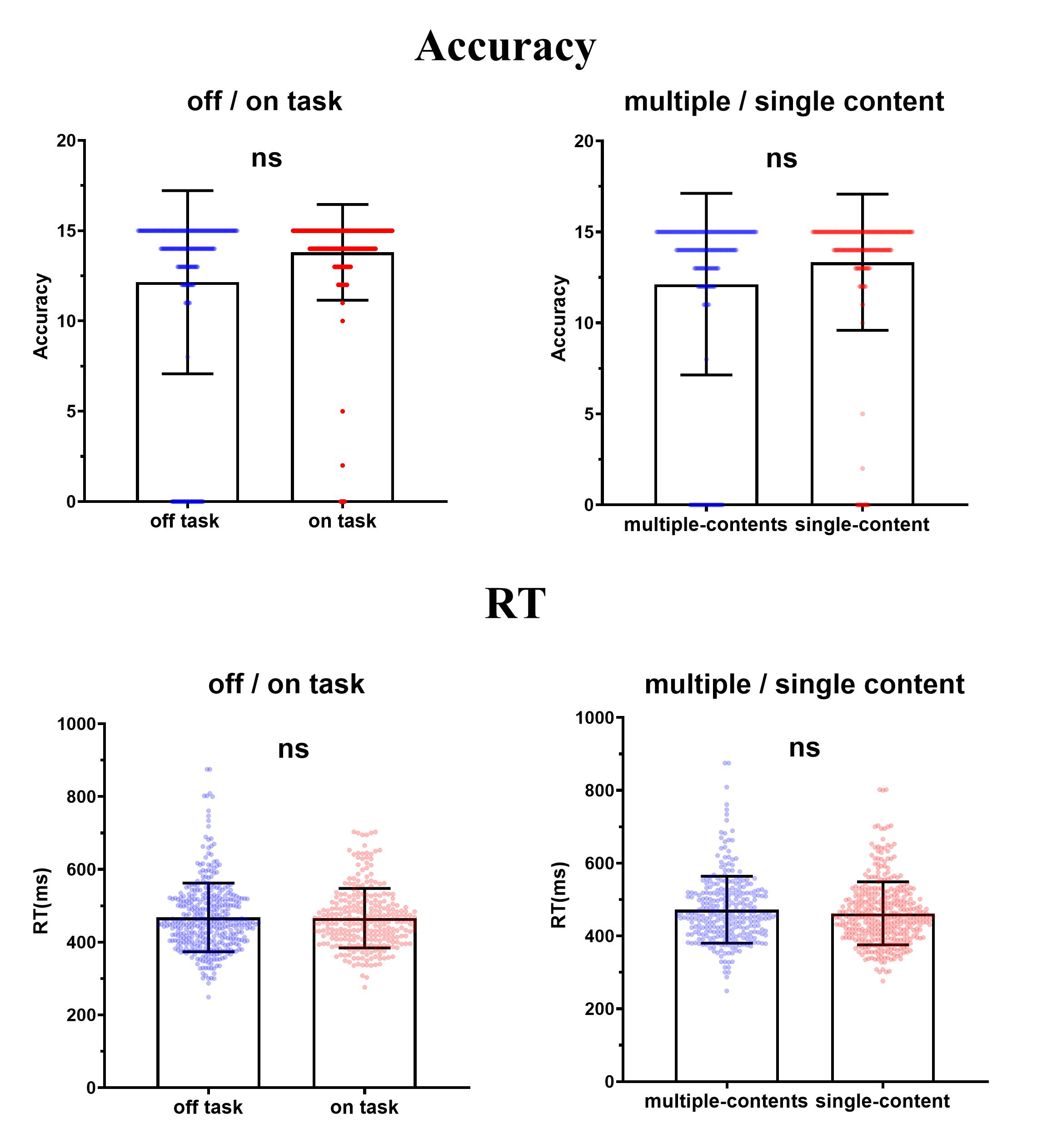




**Supplementary Figure 1 Results of accuracy and RT between off task and on task, multiple contents and single content.**

The differences between conditions were tested by LMM for RT and GLMM for accuracy. There’re no significant differences on RT and accuracy.

Error bar mean SD; ns: no significance

**Supplementary Figure 2 FS and FP during different thought types (off task, on task, multiple contents, single content)**

a): FS during different thought types. There are significant differences between off- / on- thought and multiple- / single- content during post-stim relative values on both alpha and theta bands.

b): FP during different thought types. Significance were not found on both frequency bands.

**: p<0.01; *: p<0.05; ns: no significance


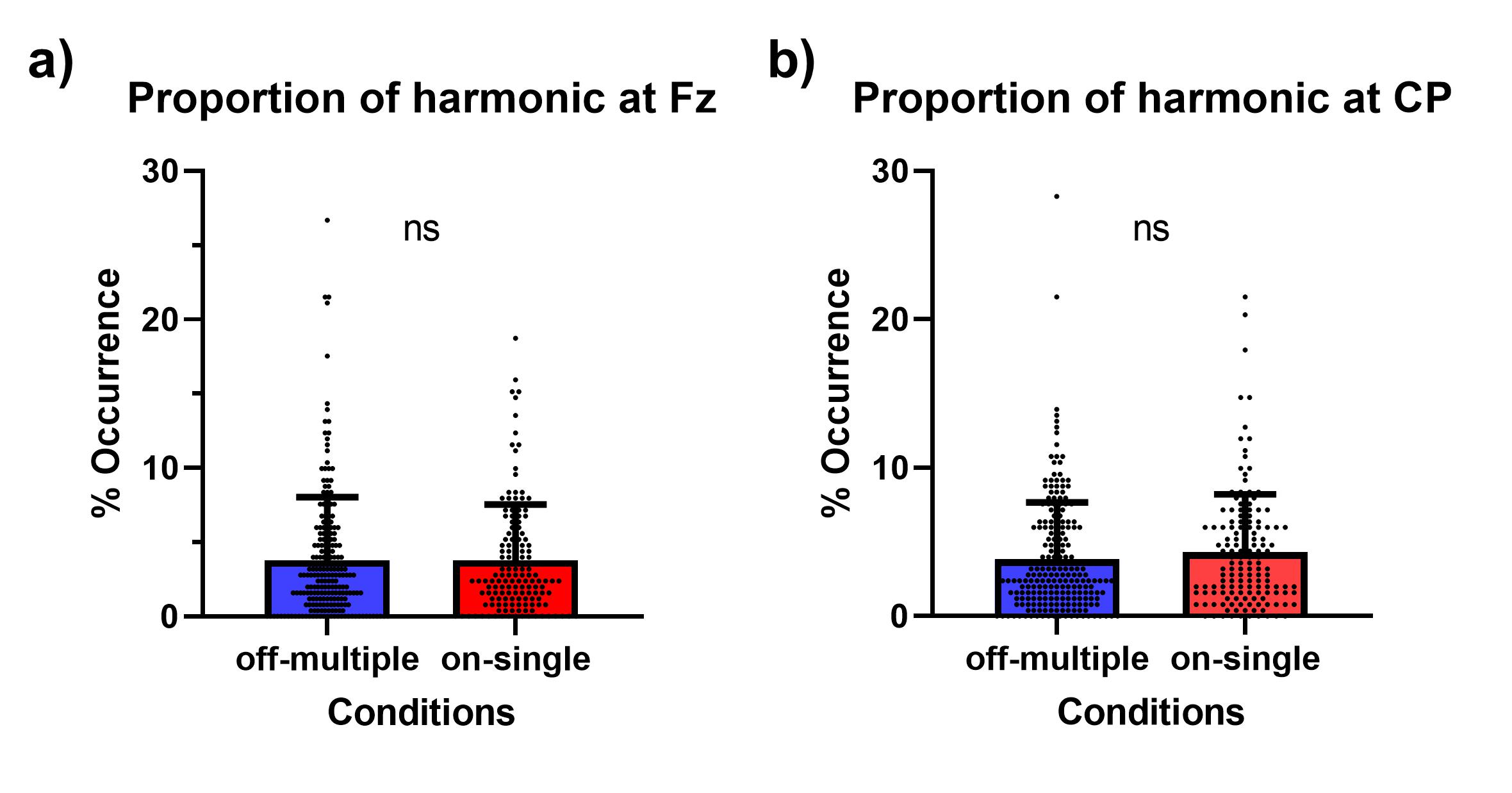


**Supplementary Figure 3 T-test between off-multiple and on-single frequency harmonic on Fz and CPz**

There are no significant differences between the proportion of harmonic of off-multiple and on-single on both a) Fz and b) CPz

Error bar mean SD; ns: no significance


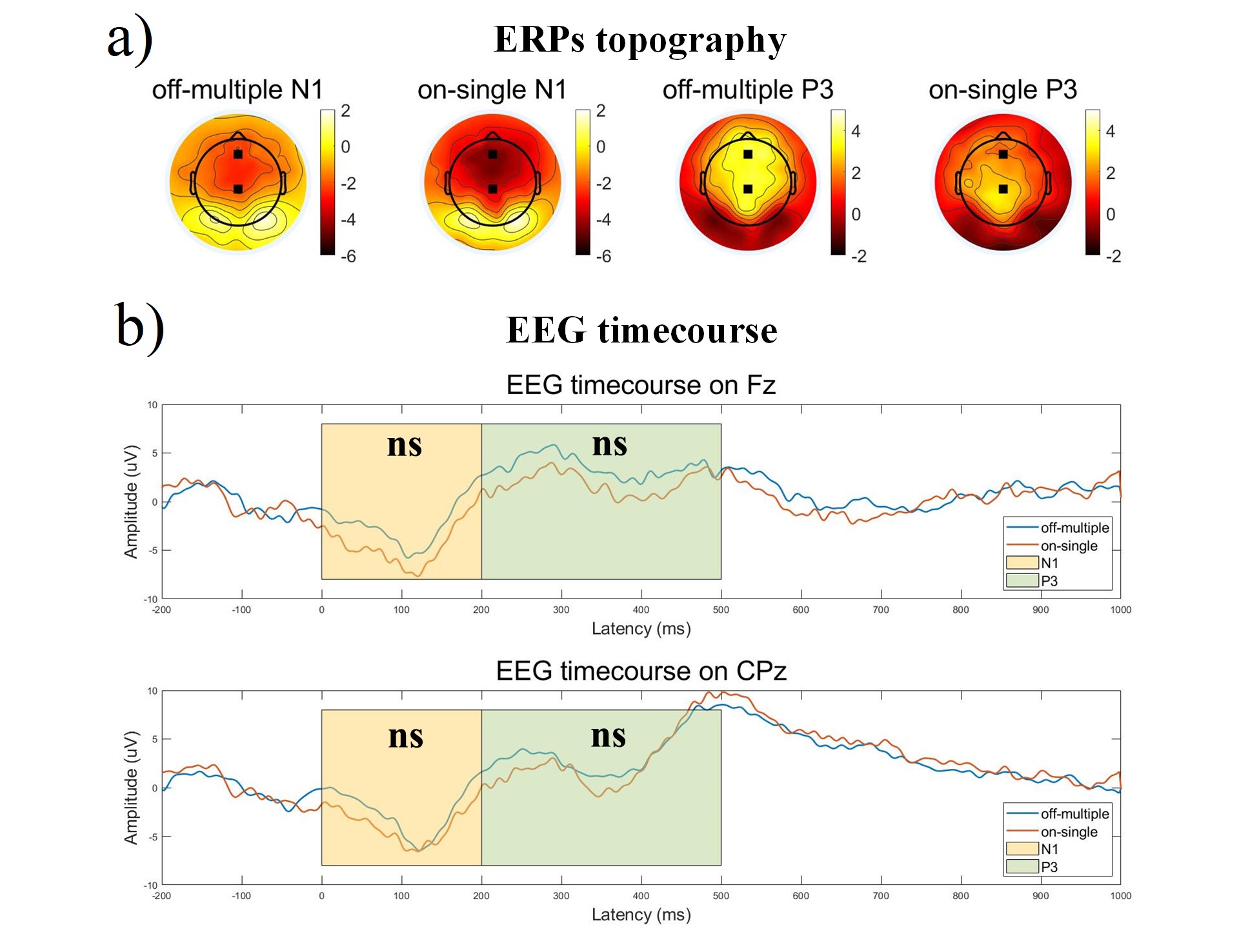


**Supplementary Figure 4 ERP results**

a). Topography of ERP components.

b). The EEG timecourses on electrodes Fz and CPz. The LMM test on N1 (the component from 0 ms to 200 ms) and P3 (the component from 200 ms to 500 ms) between off-multiple and on-single on both Fz and CPz were applied. No significant difference were found on both components.

ns: no significance


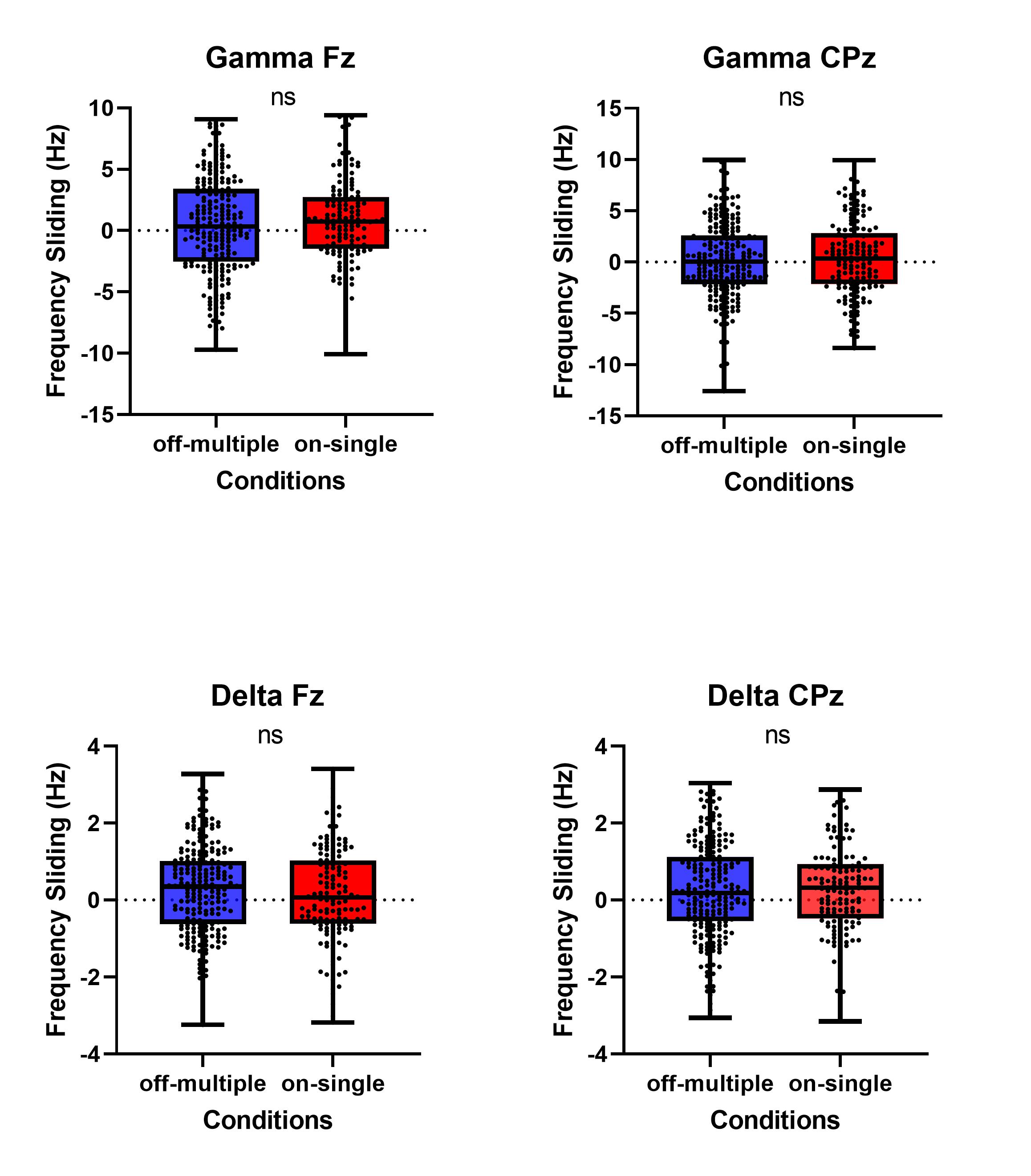


**Supplementary Figure 5 delta and gamma FS results**

FS on delta (3 Hz – 4 Hz) and gamma (30 Hz – 40 Hz) were analyzed on both Fz and CPz electrodes. The results show no significant differences on delta and gamma.

ns: no significance

**Stimuli Information**

**Standard stimuli：**

A, B, D, E, F, G, H, I, J, M, N, Q, R, T, Y

**Deviant Stimuli (Targets)：**

a, b, d, e, f, g, h, i, j, k, n, q, r, t, y

**Novel Stimuli：**

Training session：З，ф，д，я，ч

Block 1 和Block 4：Bhutanese：ང，ཀ，ཁ，ག， ན；Georgian：ჰ，დ，ი，ტ，რ；Khmer：ឆ，ឹ，េ，ុ，ា 。

Block 2 和Block 5: Bhutanese：ུ， ེ，ོ，ཏ，ཐ；Georgian：ფ，გ，ჯ，კ，ლ；Khmer：ស，ដ，ថ，ហ，ញ。

Block 3 和Block6：Bhutanese：ི，ཙ，ཞ，འ，ཡ；Georgian：ზ，ც，მ，ქ，წ；Khmer：ឋ，ខ，វ，ប，ន。
