# Supplementary material for: Alpha and theta peak frequency track on- and off-thoughts
Source: Commun Biol. 2022 Mar 7;5:209. doi: 10.1038/s42003-022-03146-w (PMC8901672; doi:10.1038/s42003-022-03146-w)
Supplement: Supplementary file 2 — Description of Additional Supplementary Files [file 42003_2022_3146_MOESM2_ESM.pdf]

Description of Additional Supplementary Files

File name: Supplementary Data 1

Description: Dataset for Figure 2

File name: Supplementary Data 2

Description: Dataset for Figure 4

File name: Supplementary Data 3

Description: Dataset for Figure 5

File name: Supplementary Data 4

Description: Dataset for Figure 6

File name: Supplementary Data 5

Description: Dataset for Supplementary Figure 1

File name: Supplementary Data 6

Description: Dataset for Supplementary Figure 2

File name: Supplementary Data 7

Description: Dataset for Supplementary Figure 3

File name: Supplementary Data 8

Description: Dataset for Supplementary Figure 4

File name: Supplementary Data 9

Description: Dataset for Supplementary Figure 5
