# Supplementary material for: Alpha and theta peak frequency track on- and off-thoughts
Source: Commun Biol. 2022 Mar 7;5:209. doi: 10.1038/s42003-022-03146-w (PMC8901672; doi:10.1038/s42003-022-03146-w)
Supplement: Supplementary file 12 — Reporting summary [file 42003_2022_3146_MOESM12_ESM.pdf]

nature research

Corresponding author(s): Georg Northoff, Chang Liu

Last updated by author(s): Dec 18, 2021

Reporting Summary

Nature Research wishes to improve the reproducibility of the work that we publish. This form provides structure for consistency and transparency in reporting. For further information on Nature Research policies, see our Editorial Policies and the Editorial Policy Checklist.

Statistics

For all statistical analyses, confirm that the following items are present in the figure legend, table legend, main text, or Methods section.

n/a	Confirmed	
☐	☒	The exact sample size (n) for each experimental group/condition, given as a discrete number and unit of measurement
☐	☒	A statement on whether measurements were taken from distinct samples or whether the same sample was measured repeatedly
☐	☒	The statistical test(s) used AND whether they are one- or two-sided<br>*Only common tests should be described solely by name; describe more complex techniques in the Methods section.*
☒	☐	A description of all covariates tested
☐	☒	A description of any assumptions or corrections, such as tests of normality and adjustment for multiple comparisons
☐	☒	A full description of the statistical parameters including central tendency (e.g. means) or other basic estimates (e.g. regression coefficient) AND variation (e.g. standard deviation) or associated estimates of uncertainty (e.g. confidence intervals)
☐	☒	For null hypothesis testing, the test statistic (e.g. F, t, r) with confidence intervals, effect sizes, degrees of freedom and P value noted<br>*Give P values as exact values whenever suitable.*
☒	☐	For Bayesian analysis, information on the choice of priors and Markov chain Monte Carlo settings
☒	☐	For hierarchical and complex designs, identification of the appropriate level for tests and full reporting of outcomes
☐	☒	Estimates of effect sizes (e.g. Cohen's d, Pearson's r), indicating how they were calculated

Our web collection on statistics for biologists contains articles on many of the points above.

Software and code

Policy information about availability of computer code

Data collection	matlab 2019b, R 4.1.1, Graphpad prism 8.
Data analysis	Eprime 2.0

For manuscripts utilizing custom algorithms or software that are central to the research but not yet described in published literature, software must be made available to editors and reviewers. We strongly encourage code deposition in a community repository (e.g. GitHub). See the Nature Research guidelines for submitting code & software for further information.

Data

Policy information about availability of data

All manuscripts must include a data availability statement. This statement should provide the following information, where applicable:

- Accession codes, unique identifiers, or web links for publicly available datasets
- A list of figures that have associated raw data
- A description of any restrictions on data availability

all data are available from the corresponding author upon request.

Field-specific reporting

Please select the one below that is the best fit for your research. If you are not sure, read the appropriate sections before making your selection.

☒ Life sciences ☐ Behavioural & social sciences ☐ Ecological, evolutionary & environmental sciences

For a reference copy of the document with all sections, see nature.com/documents/nr-reporting-summary-flat.pdf

Life sciences study design

All studies must disclose on these points even when the disclosure is negative.

Sample size	Seventy right-handed adults participated in the study, which meets the requirement of 30 subjects for each group.
Data exclusions	9 were excluded as the data were not correctly recorded or got lost. Within these 61 subjects, 13 subjects whose 12 probe choices were all the same were excluded to ensure the validity of thought probes. After EEG preprocessing, 8 subjects were excluded for bad data quality (more than 50% epochs were excluded).
Replication	Our results are consistent with previous studies.
Randomization	Participants were randomly assigned to each of 2 groups (postitive emotion group and neutral emotion group).
Blinding	The investigators were not blinded to the group assignment, which will not affect the result because the investigator was totally separated from the participants and the whole experiment program was conducted automatically by the computer.

Reporting for specific materials, systems and methods

We require information from authors about some types of materials, experimental systems and methods used in many studies. Here, indicate whether each material, system or method listed is relevant to your study. If you are not sure if a list item applies to your research, read the appropriate section before selecting a response.

Materials & experimental systems

n/a	Involved in the study	
☒	☐	Antibodies
☒	☐	Eukaryotic cell lines
☒	☐	Palaeontology and archaeology
☒	☐	Animals and other organisms
☐	☒	Human research participants
☒	☐	Clinical data
☒	☐	Dual use research of concern

Methods

n/a	Involved in the study	
☒	☐	ChIP-seq
☒	☐	Flow cytometry
☒	☐	MRI-based neuroimaging

Human research participants

Policy information about studies involving human research participants

Population characteristics	Seventy right-handed adults participated in the study (32 female; age range = 18-29 years; mean age = 22.06 years, SD = 2.71 years). All had normal or corrected-to-normal vision and reported no neurological or psychiatric conditions that might affect performance.
Recruitment	Participants were recruited online from Nanjing Normal University.
Ethics oversight	Nanjing Normal University has approved the study protocol

Note that full information on the approval of the study protocol must also be provided in the manuscript.
